# Effect of pre-exercise carbohydrate availability on fat oxidation and energy expenditure after a high-intensity exercise

**DOI:** 10.1590/1414-431X20186964

**Published:** 2018-03-26

**Authors:** G.A. Ferreira, L.C. Felippe, R.L.S. Silva, R. Bertuzzi, F.R. De Oliveira, F.O. Pires, A.E. Lima-Silva

**Affiliations:** 1 Grupo de Pesquisa em Ciências do Esporte, Centro Acadêmico de Vitória, Universidade Federal de Pernambuco, Vitória de Santo Antão, PE, Brasil; 2 Grupo de Estudos em Desempenho Aeróbio, Escola de Educação Física e Esporte, Universidade de São Paulo, São Paulo, SP, Brasil; 3 Núcleo de Estudos do Movimento Humano, Departamento de Educação Física, Universidade Federal de Lavras, Lavras, MG, Brasil; 4 Grupo de Estudos em Psico-fisiologia do Exercício, Escola de Artes, Ciências e Humanidades, Universidade de São Paulo, São Paulo, SP, Brasil; 5 Grupo de Pesquisa Desempenho Humano, Universidade Tecnológica Federal do Paraná, Curitiba, PR, Brasil

**Keywords:** Excess post-exercise oxygen consumption, Fat oxidation, Carbohydrate oxidation, Diet manipulation, Aerobic metabolism

## Abstract

The aim of this study was to test the hypothesis that reduced pre-exercise carbohydrate (CHO) availability potentiates fat oxidation after an exhaustive high-intensity exercise bout. Eight physically active men underwent a high-intensity exercise (∼95% V̇O_2max_) until exhaustion under low or high pre-exercise CHO availability. The protocol to manipulate pre-exercise CHO availability consisted of a 90-min cycling bout at ∼70% V̇O_2max_ + 6 × 1-min at 125% V̇O_2max_ with 1-min rest, followed by 48 h under a low- (10% CHO, low-CHO availability) or high-CHO diet (80% CHO, high-CHO availability). Time to exhaustion was shorter and energy expenditure (EE) lower during the high-intensity exercise in low- compared to high-CHO availability (8.6±0.8 and 11.4±1.6 min, and 499±209 and 677±343 kJ, respectively, P<0.05). Post-exercise EE was similar between low- and high-CHO availability (425±147 and 348±54 kJ, respectively, P>0.05), but post-exercise fat oxidation was significantly higher (P<0.05) in low- (7,830±1,864 mg) than in high-CHO availability (6,264±1,763 mg). The total EE (i.e., exercise EE plus post-exercise EE) was similar between low- and high-CHO availability (924±264 and 1,026±340 kJ, respectively, P>0.05). These results suggest that a single bout of high-intensity exercise performed under low-CHO availability increased post-exercise fat oxidation, and even with shorter exercise duration, both post-exercise EE and total EE were not impaired.

## Introduction

It has recently been argued that a single bout of high-intensity exercise (HIE; i.e., >80% V̇O_2max_) induces similar post-exercise energy expenditure (EE) to exercise performed at a moderate intensity over a longer time (1,2). For example, Larsen et al. (1) demonstrated that post-exercise EE increased similarly after a single bout of a 4-min HIE [∼90% maximal heart rate (HR_max_) compared to a longer exercise bout at moderate intensity (47 min at 70% HR_max_)]. In that study, post-exercise oxygen consumption (V̇O_2_) remained above rest by ∼40 min in both modes of exercise, even though high-intensity exercise was ∼11 times shorter. A single bout of high-intensity exercise results also in a higher post-exercise fat oxidation than in low-intensity exercise (2). As higher fat oxidation post-exercise has an important role in exercise-induced fat loss (3), this mode of training might be an interesting approach to overcome the sedentary lifestyle associated with the lack of free time in modern life (4). Furthermore, any manipulation able to improve post-exercise fat oxidation would be an interesting approach to enhance exercise-induced health benefits (5).

Nutritional interventions by handling carbohydrate (CHO) availability has also played a critical role in post-exercise metabolism (6). Endogenous CHO availability is commonly manipulated as follows: 1) by an 8–12 h fasting period before training, reducing liver but not muscle glycogen (7); 2) by reducing CHO diet content for a few days, reducing mainly liver glycogen (8); 3) by performing a pre-exercise before the main exercise to reduce pre-exercise liver and muscle glycogen (9); 4) or by a combination of two or more of those approaches (6,9). Regardless of the different ways to manipulate CHO stores, some evidence suggests that fat oxidation over the 24-h post-exercise period is increased when exercise is performed with a certain level of CHO depletion (5,7).

While a low-CHO availability increases post-exercise fat oxidation (7), manipulating CHO availability may reduce the exercise tolerance during a HIE bout (6). As post-exercise EE is positively related to the exercise duration, any manipulation that reduces the time length of a HIE bout will directly affect post-exercise EE (6,10). Only one study has investigated this question while manipulating CHO availability. Indeed, performing a supra-maximal exercise (125% V̇O_2_max) until exhaustion with low-CHO availability seems to reduce EE during the exercise, ultimately decreasing post-exercise EE by ∼26% (6). This would reduce HIE benefits because a reduced post-exercise EE may be detrimental to body mass loss (2,3). It has yet to be determined, however, whether a similar reduction in post-exercise EE with low-CHO availability happens in HIE when performed below V̇O_2max_ (i.e., submaximal intensities).

The aim of the present study was to quantify the effect of pre-exercise low-CHO availability on fat oxidation and EE after a single HIE bout performed until exhaustion. It was hypothesized that low-CHO availability would reduce time to exhaustion and post-exercise EE, but enhance fat oxidation after a HIE bout, compared with a high-CHO availability condition. To produce the greatest differences between the experimental conditions in terms of CHO availability, we manipulated endogenous CHO stores by combining a muscle glycogen-reducing protocol and diet.

## Material and Methods

### Participants

Eight physically active men [age=26.6±6.0 years, height=175.4±4.3 cm, body mass=72.9±6.5 kg, body fat=12.4±4.6%, and maximal oxygen uptake (V̇O_2max_)=46.4±6.4 mL·kg^-1^·min^-1^], participated in this study. Participants were classified as physically active according to their V̇O_2max_ (>42 mL·kg^-1^·min^-1^) (11). Participants signed an informed consent form after receiving verbal and written explanations of the experimental procedures and possible risks involved in this study. The study was approved by the Ethics Committee of the Universidade de São Paulo, São Paulo, SP, Brazil.

### Experimental design

Participants reported to the laboratory on 6 different occasions. In the first visit, anthropometric measurements were performed to determine height, body mass, and body fat. Then, participants completed an incremental test to exhaustion to determine their first (LT_1_) and second (LT_2_) lactate thresholds, as well as peak power output (PPO) and V̇O_2max_. The LT_1_, LT_2_, PPO, and V̇O_2max_ were used to determine the exercise-glycogen depletion and HIE intensities (see below). On the second visit, participants performed a familiarization protocol with the HIE procedures. Participants performed a HIE bout until exhaustion, exactly as they were to do in the experimental trial. The experimental trials were done in 2 blocks of 2 visits each, i.e., visits 3–4 and 5–6. The "blocks" of visits 3–4 and 5–6 were performed in a randomized, counterbalanced and crossover design, seven days apart to prevent any residual effect of fatigue. Briefly, in the third visit, participants performed a glycogen-depletion exercise protocol 48 h before each experimental session (∼8:00 am). This protocol was performed to ensure that all participants initiated the diet protocol (i.e., low- or high-CHO diet) with similar glycogen content. After the glycogen-depletion protocol, 50% of the participants followed a low-CHO diet over the next 48 h, while the other 50% followed a high-CHO diet over the next 48 h. In the fourth visit, which was performed 48 h after the exercising-glycogen depletion protocol, participants performed a single HIE bout until exhaustion. The same procedures were performed in visits 5 and 6, except that the participants who had followed the low-CHO diet in the first block received the high-CHO diet, and vice-versa. This combination of glycogen-depletion exercise and diet manipulation was applied to produce greater differences in endogenous CHO availability before the HIE (12). A prolonged exercise followed by a low-CHO diet is believed to maintain the levels of muscle glycogen low while a high-CHO diet after a bout of prolonged exercise is believed to restore muscle glycogen (13,14). Participants were asked to refrain from exercise, alcohol, and caffeine 48 h before each experimental session.

### Anthropometric

The anthropometric measurements consisted of height, body mass, and the thickness of three skinfolds (chest, abdomen, and thigh sites). Body density was estimated according to Jackson and Pollock (15) and then converted to body fat percentage using Siri (16).

### Incremental test

The incremental test was performed on a magnetically-braked cycle ergometer (Ergo Fit 167, Ergo-FitGmbH & Co., Germany). Participants warmed up for 5 min at 50 watts (W); thereafter, the power output was increased 20 W every 3 min until exhaustion, which was defined as the incapacity to maintain a pedal cadence above 60 revolution per minute (rpm). Blood samples (25 μL) were obtained from the earlobe 15 s prior to the end of each stage for immediate blood lactate concentration determination (YSI 1500 Sport, Yellow Springs Instruments, USA). Oxygen uptake (V̇O_2_) and carbon dioxide production (V̇CO_2_) were obtained breath-by-breath throughout the test using a metabolic cart (Quark b2, COSMED, Italy). Heart rate (HR) was measured throughout the test using a HR monitor (Polar Vantage NV, Finland). For identification of LT_1_ and LT_2_, blood lactate was plotted as a function of exercise intensity. The LT_1_ was considered as the workload corresponding to an initial lactate accumulation in the blood (17). The LT_2_ was considered as the workload corresponding to the second abrupt increase in lactate accumulation in the blood (17). LT_1_ and LT_2_ were identified by adjustment of the blood lactate-exercise intensity curve with a 3-segment linear regression (17). The first and second intercepts of the fitted curve that produced the highest R^2^ and the lowest residual sum of square corresponded to LT_1_ and LT_2_, respectively. The V̇O_2max_ was defined as the highest 30-s V̇O_2_ average obtained during the test, while the PPO was calculated as the highest power output reached during the test.

### Exercising-glycogen depletion protocol and diet manipulation

Participants performed an exercise protocol to reduce muscle glycogen stores. They performed 90 min of continuous exercise at a power output corresponding to 50% of the difference between LT_1_ and LT_2_. After a 5-min rest, they performed 6×1-min exercise bouts at 125% V̇O_2max_, with 1-min resting intervals. It has been previously shown that this protocol reduces muscle glycogen content to ∼50% of the pre-exercise value (18). Thereafter, participants followed an isoenergetic (mean±SD: 10,290±2,877 kJ/day, estimated by habitual dietary records) 48-h diet regime with 10 or 80% of CHO (low- or high-CHO availability, respectively; see Table 1). All participants were given a list with food options created by a dietitian that indicated the recommended daily energy uptake for each food group and providing the recommended daily energy uptake. To measure the adherence to prescribed diets, the participants recorded all food intake for 48 h through a food questionnaire between the exercise depletion protocol and the experimental exercise session (19). They were also asked to use household measures to improve accurate food portions (20) for which they received verbal and visual explanations. Diet records were subsequently analyzed for the macronutrient intakes using specific software (DietWin software, Brazil).

**Table 1.** Macronutrients intake during the low- and high-(CHO) diet.

	Low-CHO availability	High-CHO availability
CHO (g)	115±45	596±204
CHO (kJ)	1,917±755	9,968±3,413
CHO (%)	17±13	77±6
Fat (g)	214±110	40±3
Fat (kJ)	8,048±4,127	1,513±116
Fat (%)	60±18	12±5
Protein (g)	171±17	84±10
Protein (kJ)	2,860±284	1,411±166
Protein (%)	23±6	11±3

CHO: carbohydrate. Data are reported as means±SD (n=8).

### Experimental trial

Participants arrived at the laboratory after ∼8-h overnight fasting and rested sitting on a chair for 20 min. Then, participants performed a single HIE bout until exhaustion at a power output corresponding to 75% of the difference between LT_2_ and PPO (∼95% V̇O_2max_). Exhaustion was assumed when the participants were unable to maintain a pedal cadence above 60 rpm. After the test, participants recovered, sitting on a chair for 60 min. Blood samples (25 μL) were obtained from the earlobe immediately before, and at 1, 3, and 5 min after the HIE. During the exercise and entire recovery period, V̇O_2_ and V̇CO_2_ were measured for posterior EE and fat and CHO oxidation determination. The participants were not allowed to drink water during the exercise and recovery periods.

### Measurements

The EE during the exercise was calculated by the area under the curve of exercise V̇O_2_ using the trapezoidal method (21). The total VO_2_ (L) obtained was then converted to energy (kJ), assuming that each 1 L of O_2_ was equal to 20.92 kJ. The V̇O_2_, V̇CO_2_, and respiratory exchange ratio (RER) data after HIE were averaged into 5-min intervals, and fat and CHO oxidation rates estimated using stoichiometric equations (Equations 1 and 2, respectively), assuming that the urinary nitrogen excretion rate was negligible (22). As pulmonary V̇O_2_ and V̇CO_2_ are not reliable to estimate fat and CHO oxidation rates in a non-stable bicarbonate pool, data from the first 5 min of recovery were not included to estimate post-exercise substrate oxidation rate and EE (22). The total fat and CHO oxidized during the remaining period (i.e., from min 5 to 60) was calculated as the area under the fat and CHO oxidation rate curves, respectively. The post-exercise EE was assumed as the sum of fat and CHO oxidized during this period. The fat and CHO oxidation rates (mg/min) were converted to energy equivalents (kJ) assuming that 1000 mg of fat and CHO oxidized were equal to 37.67 and 16.74 kJ, respectively (23).

$$\text{Fat oxidation rate (mg/min)=1}\text{.67}\cdot {\text{V̇O}}_{\text{2}}-1.67\cdot {\text{V̇CO}}_{\text{2}}$$ (1)

$$\text{CHO oxidation rate (mg/min)}=4.55\cdot {\text{V̇CO}}_{\text{2}}-3.21\cdot {\text{V̇O}}_{\text{2}}$$ (2)

where, V̇O_2_ is oxygen uptake (mL/min) and V̇CO_2_ is carbon dioxide production (mL/min).

### Statistical analyses

The Smirnov-Kolmogorov test was used to check if the data had a Gaussian distribution. As all dependent variables were normally distributed, a two-way, repeated measures analysis of variance (ANOVA) was used to compare the lactate and V̇O_2_ at rest, and at the end of the exercise, RER, fat, and CHO oxidation rates, and post-exercise EE rate throughout the recovery, followed by a Tukey post-hoc test to locate any differences between conditions and time points. A paired *t*-test was used to compare the following dependent variables between low- and high-CHO diets: exercise duration; total VO_2_ consumed and EE during exercise; post-exercise EE, total CHO, and fat oxidized; and total EE (i.e., exercise EE plus post-exercise EE). Cohen's effect size was calculated and interpreted as ≤0.2=trivial, >2 and ≤0.6=small, >0.6 and ≤1.2=moderate, and >1.2=large. The level of significance was set at P<0.05. All statistical procedures were done in Statistica (StatSoft Inc.¯, version 10, USA). Data are reported as means±SD.

## Results

### Exercise-glycogen depletion protocol and diet manipulation

The mean power output during the continuous (∼70% V̇O_2max_) and supramaximal bouts (125% V̇O_2max_) for glycogen depletion was 160±50 and 288±58 W, respectively. The mean power output during the HIE was 219±50 W (∼95%V̇O_2max_). Macronutrient consumption during the diet manipulation period is shown in Table 1. An analysis of food records was used to indicate that the participants had ingested the recommended CHO content for each experimental condition.

### Physiological response to exercise

The blood lactate and V̇O_2_ at rest and at the end of exercise were similar between conditions (all P>0.05 and effect size <0.3 small). Time to exhaustion was 20±18% shorter in low- compared to high-CHO availability (P=0.04, effect size =0.8 moderate). Likewise, total VO_2_ was lower under low- compared to high-CHO availability (P=0.04, ES=0.7 moderate) (Table 2).

**Table 2.** Values for lactate and V̇O_2_ at rest and at the end of exercise, exercise time to exhaustion, and total VO_2_ consumed during the exercise.

	Low-CHO availability	High-CHO availability	P	ES
Rest lactate (mmol/L)	0.9±0.6	0.9±0.7	0.96	<0.2
End lactate (mmol/L)	7.6±1.9	8.4±1.9	0.52	0.3
Rest V̇O_2_ (mL·kg^-1^·min^-1^)	4.0±1.4	4.3±1.5	0.16	0.3
End V̇O_2_ (mL·kg^-1^·min^-1^)	41.4±8.4	43.1±8.5	0.22	0.2
Time to exhaustion (min)	8.6±2.3*	11.4±4.5	0.04	0.8+
Total VO_2_ (L)	24±10*	32±17	0.04	0.7+

Data are reported as means±SD (n=8). V̇O_2_: oxygen consumption; ES: effect size.

* P<0.05 compared to high-CHO availability (*t*-test).

+ Moderate effect size.

### Post-exercise RER, and CHO and fat oxidation

**Figure 1.**
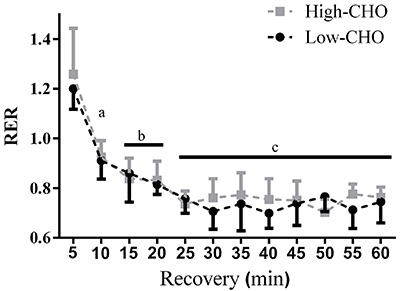
Respiratory exchange throughout the recovery of a single, high-intensity exercise bout (∼95% V̇O_2max_) performed under low- and high- carbohydrate (CHO) availability. Data are reported as means±SD (n=8). ^a^P<0.05 compared to 5 min; ^b^P<0.05 compared to 5 and 10 min; ^c^P<0.05 compared to 5, 10, 15, and 20 min (ANOVA). RER: respiratory exchange ratio.

The RER decreased up to 20 min, with no difference between conditions (main effect of time, F_(11, 77)_=44, P<0.001, Figure 1). However, RER values at 10 min were lower than 1.0 (0.92±0.06), indicating that CHO and fat oxidation rates could be estimated thereafter.

**Figure 2.**
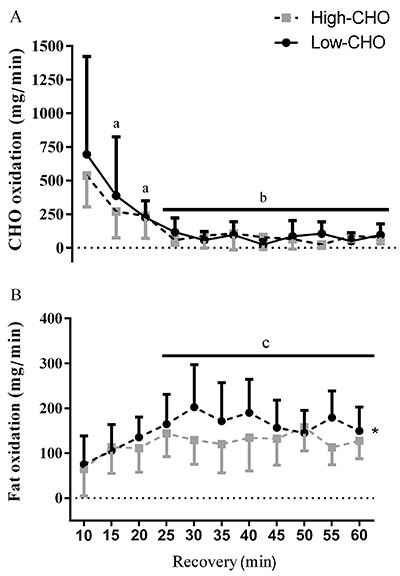
Carbohydrate (CHO) (*A*) and fat (*B*) oxidation rates throughout the recovery of a single, high-intensity exercise bout (∼95% V̇O_2max_) performed under low- and high-CHO availability. Data are reported as means±SD (n=8). ^a^P<0.05 compared to 10 min; ^b^P<0.05 compared to 5, 10, and 15 min; ^c^P<0.05 compared to 5, 10, and 15 min; *P<0.05 compared to high-CHO availability (ANOVA and *t*-test).

Post-exercise CHO oxidation rate decreased for the first 25 min and then remained stable throughout the recovery, without differences between the conditions (main effect of time, F_(10, 70)_=11.5, P<0.001, Figure 2A). Post-exercise fat oxidation rate increased for the first 25 min and remained stable throughout the recovery (main effect of time, F_(10, 70)_=3.7, P<0.001, Figure 2B). However, the fat oxidation rate was higher in the low- rather than in the high-CHO availability (main effect of condition F_(10, 70)_=6.2, P=0.04, Figure 2B). However, there was no interaction between condition × time (interaction: F_(10, 70)_=1.4, P>0.05).

**Figure 3.**
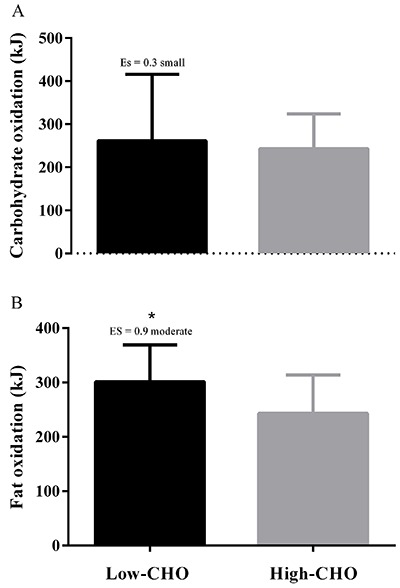
Total carbohydrate (CHO) (*A*) and fat (*B*) oxidation throughout recovery of a single, high-intensity exercise bout (∼95% V̇O_2max_) performed under low- and high-CHO availability. Data are reported as means±SD (n=8). *P<0.05 compared to high-CHO availability, moderate effect size (ES=0.9) (*t*-test).

The total CHO oxidized during recovery was similar between low- and high-CHO availability (P=0.62, effect size=0.3 small, Figure 3A), while total fat oxidized during recovery was 29±29% higher in low- rather than in high-CHO availability (P=0.046, effect size=0.9 moderate, Figure 3B).

### Energy expenditure during and after exercise, and total energy expenditure

**Figure 4.**
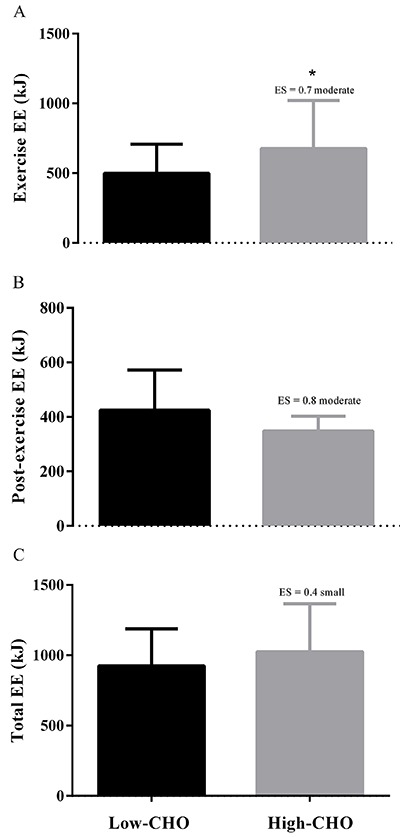
Energy expenditure (EE) during (*A*) and after (*B*) a single, high-intensity exercise bout (∼95% V̇O_2max_) performed under low- and high-carbohydrate (CHO) availability. Total energy expenditure (exercise plus post-exercise) is also shown (*C*). Data are reported as means±SD (n=8). *P<0.05 compared to low-CHO availability, moderate effect size (ES=0.7) (*t*-test).

The exercise EE was lower in low- compared to high-CHO availability (P=0.04, effect size=0.7 moderate, Figure 4A). However, post-exercise EE was similar between conditions (P=0.18, Figure 4B), although the effect size was moderate (0.8). Likewise, there was no difference for total EE (exercise EE plus post-exercise EE) between low- and high-CHO availability (P=0.34, effect size=0.3 small, Figure 4C).

## Discussion

The main finding of the present study was that HIE bout performed under low-CHO availability increased post-exercise fat oxidation rate. Even with shorter exercise duration and a lower EE exercise, both post-exercise and total EE were similar between high- and low-CHO availability. Our results suggest that reducing pre-exercise CHO availability optimizes fat oxidation after a HIE bout performed until exhaustion.

Compared with previous studies reporting reduced time to exhaustion during a HIE bout performed under reduced endogenous CHO stores (6,9), the present study showed that HIE tolerance was reduced ∼20% in low- compared to high-CHO availability. This reduced exercise tolerance might be attributed to a reduced glycogenolysis flux caused by reduced muscle glycogen (24). In fact, HIE performed at intensities near the power output eliciting V̇O_2max_ demands large ATP production via CHO oxidation, which is mainly dependent on muscle glycogen use while fat oxidation is almost nil (24,25). Further, lower exercise tolerance during a HIE with low-CHO availability might also be related to other factors such as increased exercise-induced strain, impaired excitation-contraction coupling and increased effort perception (8,24,26).

In the present study, post-exercise fat oxidation rate was increased by ∼29%. It has been proposed that beginning exercise with low-CHO content leads to a further switch in post-exercise metabolism from CHO to fat oxidation (5,7). A previous study has compared low-intensity exercise (100 min at 65% V̇O_2max_) performed before breakfast or after lunch (7). The 24 h post-exercise CHO oxidation was diminished when exercise was performed before breakfast and increased when exercise was performed after lunch, suggesting there is a change from CHO to fat oxidation with exercise beginning with depleted pool of endogenous CHO (7). Furthermore, it is largely recognized that low-CHO availability increases fat oxidation by a catecholamine-induced increase in the lipolysis, which increases free fatty acids release from the adipose tissues and consequently muscle intake (27–29). Increased fat oxidation with low-CHO availability might also be associated with an increased circulation of very-low-density lipoprotein-triacylglycerol, which may be oxidized by peripheral tissues including skeletal muscle (28,30). In fact, it has been demonstrated that all aforementioned physiological events are magnified by low-CHO availability (31,32). Our results add to these studies, showing that fat oxidation after a short-term HIE can be increased when performing with low-CHO availability.

However, it should be noted that the low-CHO availability reduced HIE duration and ultimately exercise EE. Post-exercise EE was similar between low- and high-CHO availability, although a higher fat oxidation post-exercise seems to increase slightly post-exercise EE (effect size=0.8, moderate). This led to a similar total energy expenditure (exercise + post-exercise) between conditions (effect size=0.4, small). Given that HIE with low-CHO availability did not increase the total EE, and even reduced EE during the exercise, a question arises whether increased post exercise fat oxidation after performing HIE with low-CHO availability has any practical relevance. It could be argued that any post-exercise adaptations to substrate metabolism are negated by the reduced exercise capacity reported in the low CHO trial. On the other hand, it has been previously suggested that strategies to maximize fat oxidation may be an important factor in preventing body fat accumulation, regardless of EE (2,3). In fact, it has been demonstrated that oxidation of more fat during rest is an isolated predictor of losing fat in woman engaged in an exercise program (3). Increased fat oxidation at rest explained 7% of the exercise-induced reduction in body fat mass (3). In addition, it has been reported that an increase in post-exercise fat oxidation after a single high-intensity exercise diminishes body fat deposition (2). Furthermore, increased post-exercise fat oxidation promotes an increase in several transcriptional genes associated with the muscle oxidative phenotype, increasing muscle capacity to oxidize fat (33). These findings suggest that higher rates of post-exercise whole-body fat oxidation may have an important role, regardless of the small effect on EE.

Exercise-induced disruption in homeostasis is necessary to promote health benefits associated with exercise training (4,31). As the lack of free time in modern life is a limiting factor for exercise practice and adherence (34), a single HIE bout until exhaustion becomes an alternative training model to stimulate active lifestyle (4). This model of exercise is suggested to elicit similar health benefits compared to several intermittent bouts of HIE with short intervals for recovery, despite the shorter duration of the former (4). In the present study, we showed that combining a single HIE bout performed until exhaustion with reduced pre-exercise CHO availability can maximize post-exercise fat oxidation. However, as total EE was not altered, whether this increased post-exercise fat oxidation has any impact on reduction in body fat is an open question (35). Further studies investigating if long-term training using a single bout of HIE with low-CHO availability promotes higher body fat reduction are required and may be a promising approach to weight loss reduction programs.

The main limitation of the current study was that we did not measure glycogen levels, which could be replenished by 25% for each 24-h recovery period, even under a low-CHO diet (36). Even though muscle glycogen content is partially replenished after exercise depleting muscle glycogen followed by a low-CHO diet, muscle glycogen will remain low compared to a high-CHO diet (14). Thus, two distinct conditions with different CHO availability were successfully obtained in the present study. In addition, the reduced time to exhaustion in low-CHO availability adds that CHO availability was reduced to a certain degree. Furthermore, whether the same results would occur if only diet or fasting had been manipulated is an interesting question. To the best of our knowledge, no study has compared post exercise energy expenditure and fat oxidation between glycogen-depletion exercise + diet (reducing both muscle and liver glycogen) vs only diet or fasting manipulation (reducing mostly liver glycogen), which deserves further investigation. We have also measured the adherence to prescribed diets through a food questionnaire, which may not have been accurate for food intake quantification. To improve accuracy, we used standard household measures for portions of food amount (20). To minimize any potential error in estimating total energy and macronutrients intake from diet records, the same experienced researcher performed all food register analyses. The participants were also well instructed and familiarized with the food register questionnaire. The same diet software was used to minimize the error in converting food intake into the nutrient composition and absolute amounts of energy. Finally, the food questionnaire is a recognized and valid practical method to determine food intake (19).

In conclusion, a single HIE bout performed until exhaustion with low-CHO availability reduced exercise tolerance and exercise EE, but increased post-exercise fat oxidation.

## References

[1] Larsen I, Welde B, Martins C, Tjonna AE (2014). High- and moderate-intensity aerobic exercise and excess post-exercise oxygen consumption in men with metabolic syndrome. Scand J Med Sci Sports.

[2] Yoshioka M, Doucet E, St-Pierre S, Almeras N, Richard D, Labrie A (2001). Impact of high-intensity exercise on energy expenditure, lipid oxidation and body fatness. Int J Obes Relat Metab Disord.

[3] Barwell ND, Malkova D, Leggate M, Gill JM (2009). Individual responsiveness to exercise-induced fat loss is associated with change in resting substrate utilization. Metabolism.

[4] Whyte LJ, Ferguson C, Wilson J, Scott RA, Gill JMR (2013). Effects of single bout of very high-intensity exercise on metabolic health biomarkers in overweight/obese sedentary men. Metabolism.

[5] Iwayama K, Kawabuchi R, Nabekura Y, Kurihara R, Park I, Kobayashi M (2017). Exercise before breakfast increases 24-h fat oxidation in female subjects. PLoS One.

[6] Ferreira GA, Bertuzzi R, De-Oliveira FR, Pires FO, Lima-Silva AE (2016). High-CHO diet increases post-exercise oxygen consumption after a supramaximal exercise bout. Braz J Med Biol Res.

[7] Iwayama K, Kawabuchi R, Park I, Kurihara R, Kobayashi M, Hibi M (2014). Transient energy deficit induced by exercise increases 24-h fat oxidation in young trained men. J Appl Physiol.

[8] Ferreira GA, Osiecki R, Lima-Silva AE, de Angelis-Pereira MC, De-Oliveira FR (2014). Effect of a reduced-CHO diet on the rate of perceived exertion curve during an incremental test. Int J Sport Nutr Exerc Metab.

[9] Lima-Silva AE, Pires FO, Bertuzzi R, Silva-Cavalcante MD, Oliveira RSF, Kiss MA (2013). Effects of a low- or a high-carbohydrate diet on performance, energy system contribution, and metabolic responses during supramaximal exercise. Appl Physiol Nutr Metab.

[10] Bahr R, Gronnerod O, Sejersted OM (1992). Effect of supramaximal exercise on excess postexercise O2 consumption. Med Sci Sports Exerc.

[11] De Pauw K, Roelands B, Cheung SS, de Geus B, Rietjens G, Meeusen R (2013). Guidelines to classify subject groups in sport-science research. Int J Sports Physiol Perform.

[12] Hargreaves M, McConell G, Proietto J (1995). Influence of muscle glycogen on glycogenolysis and glucose uptake during exercise in humans. J Appl Physiol.

[13] Balsom PD, Gaitanos GC, Soderlund K, Ekblom B (1999). High-intensity exercise and muscle glycogen availability in humans. Acta Physiol Scand.

[14] Arkinstall MJ, Bruce CR, Clark SA, Rickards CA, Burke LM, Hawley JA (2004). Regulation of fuel metabolism by preexercise muscle glycogen content and exercise intensity. J Appl Physiol.

[15] Jackson AS, Pollock ML (2004). Generalized equations for predicting body density of men. 1978. Br J Nutr.

[16] Siri WE (1993). Body composition from fluid spaces and density: analysis of methods. 1961. Nutrition.

[17] Ribeiro JP, Yang J, Adams RP, Kuca B, Knutten HG (1986). Effect of different incremental exercise protocols on the determination of lactate and ventilatory thresholds. Braz J Med Biol Res.

[18] Heigenhauser GJ, Sutton JR, Jones NL (1983). Effect of glycogen depletion on the ventilatory response to exercise. J Appl Physiol Respir Environ Exerc Physiol.

[19] Pears SL, Jackson MC, Bertenshaw EJ, Horne PJ, Lowe CF, Erjavec M (2012). Validation of food diaries as measures of dietary behaviour change. Appetite.

[20] Subar AF, Crafts J, Zimmerman TP, Wilson M, Mittl B, Islam NG (2010). Assessment of the accuracy of portion size reports using computer-based food photographs aids in the development of an automated self-administered 24-hour recall. J Am Diet Assoc.

[21] Artioli GG, Bertuzzi RC, Roschel H, Mendes SH, Lancha AH, Franchini E (2012). Determining the contribution of the energy systems during exercise. J Vis Exp.

[22] Frayn KN (1983). Calculation of substrate oxidation rates in vivo from gaseous exchange. J Appl Physiol Respir Environ Exerc Physiol.

[23] Darvey IG (1998). How does the ratio of ATP yield from the complete oxidation of palmitic acid to that of glucose compare with the relative energy contents of fat and carbohydrate?. Biochem Educ.

[24] Hawley JA, Leckey JJ (2015). Carbohydrate dependence during prolonged, intense endurance exercise. Sports Med.

[25] Jeppesen J, Kiens B (2012). Regulation and limitations to fatty acid oxidation during exercise. J Physiol.

[26] Correia-Oliveira CR, Bertuzzi R, Dal'Molin Kiss MA, Lima-Silva AE (2013). Strategies of dietary carbohydrate manipulation and their effects on performance in cycling time trials. Sports Med.

[27] Jeukendrup AE (2003). Modulation of carbohydrate and fat utilization by diet, exercise and environment. Biochem Soc Trans.

[28] Horowitz JF, Klein S (2000). Lipid metabolism during endurance exercise. Am J Clin Nutr.

[29] Langfort J, Pilis W, Zarzeczny R, Nazar K, Kaciuba-Uscilko H (1996). Effect of low-carbohydrate-ketogenic diet on metabolic and hormonal responses to graded exercise in men. J Physiol Pharmacol.

[30] Helge JW, Watt PW, Richter EA, Rennie MJ, Kiens B (2001). Fat utilization during exercise: adaptation to a fat-rich diet increases utilization of plasma fatty acids and very low density lipoprotein-triacylglycerol in humans. J Physiol.

[31] Frost EA, Redman LM, de Jonge L, Rood J, Zachwieja JJ, Volaufova J (2014). Interaction between dietary fat and exercise on excess postexercise oxygen consumption. Am J Physiol Endocrinol Metab.

[32] Burke LM, Hawley JA, Angus DJ, Cox GR, Clark SA, Cummings NK (2002). Adaptations to short-term high-fat diet persist during exercise despite high carbohydrate availability. Med Sci Sports Exerc.

[33] Bartlett JD, Hawley JA, Morton JP (2015). Carbohydrate availability and exercise training adaptation: too much of a good thing?. Eur J Sport Sci.

[34] Trost SG, Owen N, Bauman AE, Sallis JF, Brown W (2002). Correlates of adults' participation in physical activity: review and update. Med Sci Sports Exerc.

[35] Brinkworth GD, Noakes M, Clifton PM, Buckley JD (2009). Effects of a low carbohydrate weight loss diet on exercise capacity and tolerance in obese subjects. Obesity.

[36] Costill DL, Sherman WM, Fink WJ, Maresh C, Witten M, Miller JM (1981). The role of dietary carbohydrates in muscle glycogen resynthesis after strenuous running. Am J Clin Nutr.

